# Investigating facilitators and barriers to the routine provision of HIV PrEP in community pharmacies in London

**DOI:** 10.1186/s12913-025-12336-1

**Published:** 2025-02-25

**Authors:** Marsha Alter, Shivali Lakhani, Aos Alaa, Manisha Karki, Eva Riboli-Sasco, Austen El-Osta

**Affiliations:** 1 School of Public Health, Imperial College, London, UK; 2 Self-Care Academic Research Unit (SCARU), School of Public Health, Imperial College, London, UK

**Keywords:** Community pharmacy, Pharmacy, PrEP, HIV, Sexual health

## Abstract

**Background:**

The United Kingdom’s (UK) integration of Pre-exposure Prophylaxis (PrEP) into community pharmacies presents an alternative avenue for supporting Human Immunodeficiency Virus (HIV) prevention. Despite its effectiveness, PrEP's accessibility remains hindered by various barriers within community settings. The aim of this study was to explore the perspectives of pharmacy team members regarding the barriers and facilitators to the routine provision of PrEP in community pharmacies in the UK, as well as any recommendations for mitigating these challenges.

**Methods:**

Exploratory mixed-method cross-sectional study utilising an online survey and semi-structured interviews with community pharmacists and non-pharmacist team members across the North Thames area of London, England.

A convenience sample of 110 pharmacy team members participated in the study, including both pharmacists and non-pharmacists. Two pharmacy technicians and eight pharmacists took part in semi-structured interviews.

Data collection involved a cross-sectional online survey and semi-structured interviews. The survey collected data such as demographic characteristics, knowledge, and attitudes towards PrEP provision, while interviews explored in-depth perceptions, experiences and recommendations.

**Results:**

A significant proportion of respondents expressed a lack of confidence and knowledge regarding PrEP, with training identified as a critical need for facilitating PrEP provision. Additionally, the study highlighted the potential of community pharmacies to increase accessibility of PrEP due to their geographical reach and the trust placed in pharmacists.

**Conclusion:**

The study demonstrates an interest from community pharmacies in London in providing a commissioned PrEP supply service. However, this would need to be in conjunction with training programmes and public health campaigns to equip community pharmacies for effective PrEP provision. Enhancing pharmacists' competencies and raising public awareness could significantly support the current HIV prevention strategies in the UK.

**Supplementary Information:**

The online version contains supplementary material available at 10.1186/s12913-025-12336-1.

## Background

Pre-exposure prophylaxis (PrEP) is the use of antiretroviral drugs (prior to exposure) to help reduce the risk of contracting the Human Immunodeficiency Virus (HIV).

PrEP may either be taken orally, (e.g., tenofovir plus emtricitabine), by long-acting injectable (Cabotegravir) or vaginal ring (Dapivirine) [1]. Oral PrEP based on tenofovir disoproxil fumarate (TDF) is advised by the World Health Organisation (WHO) for people at substantial risk of acquiring HIV infection as an additional preventive option, as part of comprehensive prevention [1]. PrEP is highly effective when taken as prescribed [2]. Its effectiveness in preventing infection with HIV in high-risk patients such as men who have sex with men (MSM) [3], heterosexual couples with one HIV-positive partner [4] and injecting drug users (PWID) [5] has been validated by numerous trials. The WHO systemic review and analysis of studies, updated in 2016, found that PrEP is effective at reducing the risk of acquiring HIV regardless of age, sex, drug regimen or potential mode of transmission. However, the level of protection was strongly correlated with adherence [6]. Globally, countries are at varying stages of PrEP uptake and data compiled by the Global PrEP Network shows that oral PrEP use has been increasing globally with over 600 000 people across 76 countries receiving oral PrEP at least once in 2019 [7].

In the UK, the use of antiretroviral therapy by people who are HIV positive to both prevent and treat HIV infection (treatment as prevention [TasP]) was approved by NHS England in 2015 [8]. In 2017, NHS England announced funding for the 3-year PrEP Impact implementation trial to address outstanding questions including the need for, uptake of and duration of PrEP. In March 2020, UK Health Secretary Matt Hancock announced that PrEP would be made available free on the National Health Service (NHS) to those at elevated risk of HIV infection. Local authorities were to receive £16 million in 2020–21 from the UK Department of Health and Social Care to deliver preventive HIV treatment through local sexual health clinics [9]. In 2024, PrEP can now be obtained free of charge via sexual health clinics providing HIV services in England, Scotland, and Wales, and via Genito-Urinary Medicine (GUM) clinics in Northern Ireland. It is also possible to purchase PrEP privately via the internet. According to PrEPWatch, in July 2024, there were an estimated 98,404 PrEP users in the UK [10].

Because NHS-funded PrEP is available via a limited number of designated sexual health clinics, clients may have to travel long distances to obtain PrEP, which is impractical and costly and limits timely access to prophylactic therapy. However, community pharmacies are typically within a 15–20-min walk for most urban populations [11] and their longer opening hours offer greater accessibility than other sexual healthcare providers. However, there is no current availability of PrEP via pharmacies.

While online alternatives offer another solution, postage may be unreliable and indiscreet. The added benefit of a community pharmacy PrEP supply service, would be the chance of a consultation with a Community Pharmacist (CP) which could include discussion about concurrent medicines, self-care lifestyle advice, and is in line with the NHS England “make every contact count” consensus statement updated in 2017 [12].

When considering the potential advantage of Community Pharmacy lead clinics, numerous studies globally have shown that HIV Clinical Pharmacist interventions and clinical care activities significantly improve medication adherence and virologic outcomes in HIV-positive individuals [13]. It has been reported that in a variety of healthcare settings, pharmacists’ direct patient care significantly improves patient’s knowledge, medication adherence and quality of life [14] and a study by Ahmed et al. in 2022 found that poor adherence correlated to a higher number of complications, treatment failure, and cross-resistance to other anti-retroviral (ART) medications [15]. Studies in the United States of America (USA) have indicated the possible ease of integrating PrEP services into locations where HIV testing and linkage to care is already extant [16, 17], evening and weekend hours of pharmacy operation, pharmacists’ ability to prospectively review medication refill gaps to detect non-adherence and to provide adherence counselling [17], and partnerships with other entities (e.g. health departments or community organisations) to optimise reach to at-risk populations [14, 16, 17].

Although this example may not relate directly to models of HIV care by Pharmacists in the UK, consideration of expanding commissioned community pharmacy sexual health services, especially those which offer HIV testing and signposting to PrEP services (such as that in the borough of Haringey in London, England) [18], could improve patient education on PrEP and even supply as appropriate. With their ease of access for most communities in England, expanding portfolio of available clinical services, community pharmacists are in an ideal position to promote the safe use of PrEP, and could help abate PrEP non-adherence, important in reducing the risk of contracting new HIV infection [6].

Despite these indications, there is scant research on the facilitators for and barriers to the routine provision of PrEP in the community pharmacy landscape in England. The aim of this study was to investigate the perspectives of community pharmacy team members regarding extant barriers and the facilitators for the routine provision of HIV PrEP in community pharmacies in the UK. We also sought to identify key recommendations from the ‘front line’ pharmacy team members that could be considered to mitigate barriers to the streamlined provision of PrEP in this setting.

## Methods

### Aim

To understand pharmacist and non-pharmacist team members' knowledge, attitudes, perception, and opinions relating to barriers and facilitators to the routine provision of HIV PrEP in the community pharmacy setting.

### Study design

The study adopted a mixed-method approach using a cross-sectional online survey and semi-structured interviews from a convenience sample of pharmacy staff, including Community Pharmacists and non-pharmacist team members in London.

### Funding

The study was partially funded by the National Institute of Health and Care Research (NIHR).

### Ethics

Ethical approval was gained from the Imperial College Research Ethics Committee (ICREC) for the study “Investigating facilitators and barriers to the routine provision of HIV PrEP in community pharmacies in London” (ICREC #21IC6934). This arm of the project investigates the views of community pharmacy teams. There is a concurrent investigation of public views relating to a Community Pharmacy PrEP supply service which is covered by the same ethics approval, and which will be reported separately.

Consent to participate was gained by survey respondents and interviewees.

The study adhered to the principles of the Declaration of Helsinki.

### Study documents

Participant information documents reference recruitment groups for both the pharmacy and public surveys and interviews. Participants for the Pharmacy Study were recruited from North-Central London (NCL) and North-West London (NWL) with no further demographic targeting.

### Electronic survey

Questions for the survey were discussed with the study team. The study team-based questions on a knowledge of community pharmacy workflow and current services, with the aim of capturing respondent views relevant to a future community pharmacy PrEP supply service. The questions were designed to understand the current service landscape, perceived competencies, motivation, upskilling, and support required to provide a PrEP supply service. It was hoped to achieve at least 100 survey responses to obtain a range of viewpoints.

The voluntary survey could be accessed via a smartphone or personal computer and included 25 questions spread over many screens (Supplementary File 1). The survey can be accessed using this link: https://imperial.eu.qualtrics.com/jfe/form/SV_5jOgKj4szkJV1fo.

Before it was released, the usability of the online survey was piloted by the research team and contacts at Imperial College, London. Responses were not relevant to the study and therefore were not saved.

Participants were asked to affirm that they consented to take part in the eSurvey in the first question. Respondents were asked questions about their gender, age, race, job title and field, and the first part of the pharmacy postal code they are affiliated with, among other demographic characteristics. The pharmacy identifying code was also collected. Participants identifying details were not collected unless they consented to supply an email address to receive an Amazon voucher as an acknowledgement of their time spent on the survey or if they wished to participate in follow-up interviews.

Participants also had the option to review their responses before submitting the survey. The knowledge and attitudes of the pharmacy team towards the routine provision of PrEP in the community pharmacy setting were appraised through a number of questions. The survey included questions on sexual health services at their pharmacy, their personal knowledge of HIV and PrEP, their attitude towards providing PrEP in their pharmacy, and the perceived barriers towards PrEP in community pharmacy. Respondents had the option to choose "no opinion" to withhold their response. These responses were considered missing in all analyses (listwise exclusion); however, as the percentage of missing data was so low (< 1.5%), the data were not imputed [19, 20].

The link to the electronic survey was published and made available on the Imperial College Qualtrics platform between 28 October 2022 and 3 August 2023 (10 months).

The study team sent out invitation emails to those who could be eligible. The link to the survey, the Participant Information Sheet (PIS), together with other study materials were distributed via five Local Pharmaceutical Committees (LPCs) in the North Thames area of London. The PIS contained details on the objectives of the study, the safeguarding of participants' personal information, their freedom to discontinue participation at any moment, the types of data recorded, where they were kept, and how long they were kept there, the identity of the investigator, the study's purpose, and the duration of the survey. Only the research team had access to the eSurvey results, which were kept on the secure database of Imperial College London.

### Personal interviews

The interview questions expanded on the survey questionnaire, to open a conversation on the perceived barriers and facilitators to the supply of PrEP from community pharmacy. The research team discussed the questions (face validity) before developing an interview guide that was tested by staff contacts at Imperial College, London, and improved. The purpose of the guide was to offer organisation and direction. Test responses were not saved.

The author’s personal networks of pharmacists as well as respondents to the online survey who consented to being contacted for an interview were approached with ethically approved study information including the participant information sheet and an online consent form. The aim was to achieve at least 10 interviews with pharmacists and other members of the pharmacy team to have a range of viewpoints.

Potentially eligible participants who consented to be interviewed were provided with study information including participant information sheet describing the study's aim, the identity of the interviewers, the duration and location of the interview and the duration of data storage. Interviewees were advised not to answer any questions they felt uncomfortable answering, and that they were free to leave the interview at any time without providing an explanation. All participants provided consent to the publication of their anonymised responses. Ten one-to-one interviews were carried out face-to-face or by telephone by MA and SL during July and August 2023. The interviewers (MA and SL) knew four of the participants prior to the interview.

Participants were made aware that any quotes taken verbatim might be used to highlight important concepts would remain anonymous. The open-ended, semi-structured interview questions were intended to delve further into the experiences, attitudes, and viewpoints of the participants. There were probing questions posed, and people were invited to give further thoughts and remarks. Specifically, our objectives were to further explore the respondents’ views on the barriers to and facilitators for the introduction of PrEP in community pharmacy, with a specific focus on the professional competencies needed, as well as recommendations regarding ways to raise awareness of PrEP, especially among minority groups (see Supplementary file 2 for semi-structured interview guide).

Interviews were conducted with just the participant and the interviewer present. Without pausing or ending the interview, every interviewee finished it and responded to every question. When no more information was forthcoming, the interview was ended. Contextual problems that were pertinent were documented using field notes. The interviews ranged from ten to 20 min. Interviewees did not receive remuneration or compensation for their participation in this part of the study.

### Data analysis

Survey responses were summarised using frequencies and percentages. Descriptive analysis was performed using SPSS (Statistical Package for Social Sciences) version 28.0.1. The Checklist for Reporting Results of Internet eSurveys (CHERRIES) was used to guide reporting [21].

Contextual data from personal interviews were analysed according to the principles of practical thematic analysis [22].

The themes were derived from the data by reading and coding transcripts then checked against the interview guide, study objectives and quantitative findings. This resulted in the development of a set of major themes identified either as barriers, facilitators or recommendations. The study team did not discuss the findings with participants but was keen to share publications with anyone who expressed interest. The Consolidated Criteria for Reporting Qualitative Research (COREQ) were used to guide reporting [23, 24].

### Patient and public involvement

This study did not have involvement from patients or members of the public.

## Results

We collected quantitative data from 110 participants between October 2022 to March 2023, of which 88 were pharmacists and 22 indicated as non-pharmacists. The specific role of the non-pharmacist team members was not clarified. Emails were sent to 700 pharmacies, with some pharmacies returning survey responses from more than one team member. We also collected contextual data from personal interviews with 10 respondents, between July and August 2023. The results are presented in the same order.

### ESurvey—quantitative

#### Respondent characteristics

The electronic survey captured full responses from 110 respondents who were either registered pharmacists or other members of the pharmacy team from the North-Central and North-West areas of London (Table 1). All respondents completed the survey, and all responses were analysed. Nearly two thirds (60.9%) of respondents were male, and the majority (68.2%), were Asian/ Asian British, or worked in the independent community pharmacy sector (82.7%). Less than a quarter (21.8%) worked in a pharmacy multiple or retail group of pharmacies. Only 7.3% of respondents reported being independent prescribers. The detailed characteristics of the respondents of the survey are shown in Table 1.

**Table 1:** Respondent characteristics (*n* = 110)

	**N**	**(%)**
**Age (*n* = 109)**		
20–29	17	(15.6)
30–39	42	(38.5)
40–49	21	(19.3)
50–59	14	(12.8)
60–69	13	(11.9)
70–79	2	(1.8)
**Gender (*n* = 110)**		
Male	67	(60.9)
Female	43	(39.1)
**Ethnicity (*n* = 107)**		
Asian/Asian British	73	(68.2)
Black/African/Caribbean/Black British	5	(4.7)
Mixed/multiple ethnic groups	3	(2.8)
White	19	(17.8)
Other	7	(6.5)
**Pharmacists (*n* = 110)**		
Pharmacists	88	(80.0)
Non-pharmacist team member	22	(20.0)
**Role (*n* = 134) **^**a**^		
Community pharmacist (independent)	91	(82.7)
Community pharmacy (multiple)	24	(21.8)
GP practice-based pharmacist	4	(3.6)
Hospital pharmacist	1	(0.9)
Independent prescriber	8	(7.3)
PCN pharmacist	1	(0.9)
Other	5	(4.5)

^a^ = multiple choice question (respondents may have multiple roles, percentage calculated as respondents answering for each role related to total number of respondents)

### Provision of sexual health services in the community pharmacy setting

The results of the full survey are shown in Table 2. Only 30 respondents (27.8%) reported that their pharmacy offered sexual health services including emergency hormonal contraception (26.8%), signposting (23.7%), free condom distribution (17.5%), chlamydia screening/treatment (11.3%), STI testing (11.3%), and HIV testing (9.3%). Of the 30 pharmacies that offered sexual health services, 30.0% answered as providing level 1 (for the purpose of this study this tier is screening), 10.0% answered as providing level 2 (testing), and 23.3% provided answered as providing the fuller level 3 (treating) sexual health services.

**Table 2:** Results of electronic survey (*n* = 110)

	**N**	**(%)**
**Does your pharmacy provide sexual health services? (*n* = 108)**		
Yes	30	(27.8)
No	78	(72.2)
**Which level of sexual health services does your pharmacy provide? (*n* = 30)**		
Level 1 (screening)	9	(30.0)
Level 2 (testing)	3	(10.0)
Level 3 (treating)	7	(23.3)
Unsure	11	(36.7)
**What sexual health services does your pharmacy provide? (*n* = 97)**		
Chlamydia screening / treatment	11	(11.3)
Emergency hormonal contraception	26	(26.8)
Free condom distribution	17	(17.5)
HIV testing	9	(9.3)
Signposting	23	(23.7)
STI testing	11	(11.3)
^**a**^**Which of the following groups are at an elevated risk of HIV?**		
Ethnic minorities	49	(45.4)
Injecting drug users	96	(88.9)
Men who have sex with men	89	(82.4)
Sex workers	87	(80.6)
Young people (aged 20–24)	57	(52.8)
Other	2	(1.9)
^**a**^**What would you likely do if a patient requested PrEP in your pharmacy?**		
Signpost to an STI clinic	106	(96.4)
Suggest they proceed with a HIV test	22	(20.0)
Suggest they purchase PrEP from safe online websites	7	(6.4)
**Would you know where to signpost individuals? (*n* = 109)**		
Yes	101	(92.7)
No	8	(7.3)
**If you do not provide any sexual health services, what is the reason? (*n* = 93)**		
Need more training	33	(35.5)
Not needed where I work	18	(19.4)
Ethical / religious reasons	1	(1.1)
Unsure/ I am not the decision maker	27	(29.0)
Other	14	(15.1)
**Have you ever heard of PrEP to prevent HIV? (*n* = 110)**		
Yes	73	(66.4)
No	37	(33.6)
**If you are an independent prescriber, or are working towards an IP qualification, would you consider prescribing PrEP? (*n* = 26)**		
Yes	20	(76.9)
No	6	(23.1)
**It would be appropriate if individuals can access PrEP from the community pharmacy setting (*n* = 109)**		
Disagree	6	(5.5)
Neither agree nor disagree	17	(15.6)
Agree	86	(78.9)
**I feel confident to provide private/commissioned sexual health services in my pharmacy (*n* = 109)**		
Disagree	14	(12.8)
Neither agree nor disagree	37	(33.9)
Agree	58	(53.2)
**I feel confident to discuss issues regarding patients’ sexual health (*n* = 109)**		
Disagree	9	(8.3)
Neither agree nor disagree	26	(23.9)
Agree	74	(67.9)
**I feel confident to discuss HIV health issues/services with a patient (*n* = 109)**		
Disagree	17	(15.6)
Neither agree nor disagree	30	(27.5)
Agree	62	(56.9)
**I would feel more comfortable/confident providing services if I had more training or support (*n* = 109)**		
Disagree	5	(4.6)
Neither agree nor disagree	10	(9.2)
Agree	94	(86.2)
**Individuals will be comfortable to receive health information from their local community pharmacy regarding PrEP (*n* = 108)**		
Disagree	3	(2.8)
Neither agree nor disagree	21	(19.4)
Agree	84	(77.8)
**Making PrEP available in the community pharmacy setting will raise awareness & demand towards PrEP in the community (*n* = 109)**		
Disagree	2	(1.8)
Neither agree nor disagree	11	(10.1)
Agree	96	(88.9)
**I routinely signpost to STI clinics (*n* = 108)**		
Disagree	25	(23.1)
Neither agree nor disagree	33	(30.6)
Agree	50	(46.3)
**I feel comfortable signposting patients to HIV & sexual health services (*n* = 110)**		
Disagree	9	(8.2)
Neither agree nor disagree	17	(15.5)
Agree	84	(76.4)
**Would you consider offering a commissioned service to supply (*n* = 110)**		
Yes	83	(75.5)
No	2	(1.8)
Unsure/ not the decision maker	25	(22.7)
^**a**^**What would prevent you from offering PrEP in pharmacy?**		
Insufficient staff levels	43	(39.1)
Require more training	81	(73.6)
Time needed to counsel individuals	49	(44.5)
Uncomfortable discussing sexual matters	5	(4.5)
Other	8	(7.3)

^a^ = multiple choice question (respondents could select more than one answer. The percentage is the number of answers for each option related to the total number of respondents (110) for each line)

When asked about the usual course of action taken when a service user requested PrEP in the local pharmacy, 96.4% of respondents said they would signpost the patient to an STI clinic, 20.0% would suggest an HIV test kit, and 6.5% would suggest they purchase PrEP from safe online websites.

### Pharmacy team knowledge of HIV and PrEP

When asked about which individuals would be deemed at higher risk of contracting HIV, 88.9% of our respondents thought injecting drug users were at a high risk of contracting HIV, compared to 82.4% for MSM, 80.6% for sex workers, 52.4% for young people (aged 20–24) and 45.4% for ethnic minorities; Table 2 The majority (92.4%) of respondents stated that they knew where to signpost individuals when they needed a sexual health clinic, whereas 7.3% did not know where to signpost. Remarkably, only 66.4% of respondents had heard of PrEP to prevent HIV, with the remaining 33.6% lacking this awareness.

### Pharmacy team competencies to provide a sexual health, HIV and PrEP service

Over half (53.2%) of respondents felt confident in providing private/commissioned sexual health services in their pharmacy (Table 2). Over two-thirds (67.9%) felt confident to discuss issues regarding patient’s sexual health, whereas only 56.9% agreed they felt confident to discuss HIV health issues/services with a patient. Over three-quarters (76.4%) felt comfortable signposting patients to HIV and sexual health services, although only 46.3% routinely signposted patients/clients to STI clinics.

### Views, knowledge, and awareness of pharmacy team members on HIV and PrEP

The majority respondents (78.9%) agreed that it would be appropriate if individuals could access PrEP in the community pharmacy setting and 88.9% felt that making PrEP available in the community pharmacy setting would raise awareness and demand for PrEP in the community. (Table 2). Three quarters (75.5%) stated that they would consider offering a commissioned service to supply PrEP in the community pharmacy setting. The majority (79.6%) of respondents who were independent prescribers (IP), or working towards an IP qualification, reported they would consider prescribing PrEP.

### Barriers to the provision of PrEP in the community pharmacy setting

When asked about barriers to the provision of PrEP in their clinic, 35.5% reported that they needed more training, 19.4% stated that it was not necessary in their place of work, 29.0% were unsure or were not the decision-makers and 1.1% stated it was for ethical reasons. Most respondents (86.2%) agreed that they would feel more comfortable/confident providing sexual health services if they had more training or support (Table 2).

When asked about what would prevent them from offering PrEP in the community pharmacy setting, perceived barriers included the current lack of training (73.6%), reported the additional time needed to counsel individuals (44.5%), the perceived need for additional staff to administer PrEP as part of a specially designed service (39.1%), Only discomfort in discussing sexual matters (4.5%).

### Personal interviews—qualitative

#### Participant characteristics

Two pharmacy technicians and eight pharmacists (six female and four male) consented to take part in personal semi-structured interviews, lasting between 10 and 20 min. All interviewees worked in Community Pharmacy settings which provided sexual health services such as contraception, sexual health advice with screening (verbal symptom checking during consultation) and signposting to other sexual health services as appropriate.

Key themes are presented in Table 3 below.

**Table 3:** Perceived barriers, facilitators and recommendations identified by respondents

Category	Level	Theme
**Barriers**	Pharmacy	Lack of familiarity & specific training on HIV and PrEP
		Limited human resources
		Expected increased workflow
		Lack of confidence regarding this specific health topic
	General public	Lack of awareness regarding HIV and PrEP
		Stigma
		Cultural sensitivities
**Facilitators**	Pharmacy	Positive attitude of staff regarding provision of PrEP
		Approachability and accessibility of pharmacy staff
		Ability to provide personalised advice to client based on lifestyle & the current medications they are on
	Pharmacy / Public	Geographical accessibility of pharmacies
**Recommendations**	Pharmacy	Accredited training focused on provision of PrEP
	General public	Educational campaigns for all
		Targeted campaigns for disadvantaged & minority groups

### Barriers

Respondents identified several barriers to the provision of PrEP in community pharmacies, stemming from both the pharmacy team and the end-users' perspectives. In the former, the main barrier identified by all interviewees was a lack of familiarity and current lack of specific training regarding HIV and PrEP. This resulted in a lack of confidence in pharmacy team members to be involved in the supply of PrEP. Interviewees expressed that this could be addressed by the provision of specific training about HIV, and PrEP including dosage and modes of use. Limited staffing capacities and expected increased workflow associated with the provision of PrEP as an additional service were also raised as possible barriers, as were concerns about how a commissioned PrEP service would be funded and the need for a reliable and confidential record-keeping system.

*“…lack of community pharmacy training, lack of confidence of team members (not just pharmacists) speaking to the client about PrEP, training needed for whole team (regarding PrEP and HIV).”* (Female Pharmacist p2).

*“…education (for public and pharmacy teams), funding and training needed for a new PrEP service, the biggest issue is money to facilitate”* (Female Pharmacist p*8*).

Other perceived barriers centred around the lack of public awareness and cultural sensitivities regarding the use of HIV preventive medicine. In addition, pharmacists acknowledged that this is a sensitive topic which might require additional privacy to be adequately, comfortably, and safely discussed.

*“People in different communities may have issues sharing experiences and not wanting people to know. Privacy would be needed at the Pharmacy.”* (Female Pharmacist p8).

*“This is a taboo subject for some cultures and families”* (Female Pharmacist p9).

Finally, one respondent voiced concern that increasing access to PrEP could promote promiscuity stating that a community pharmacy PrEP supply service.

*“*…*may possibly aid in reducing new incidence of HIV infection, but I’m concerned that this potentially may increase promiscuity”* (Male Pharmacist p5).

### Facilitators

When prompted, all interviewees spoke positively about the potential benefits of a Community Pharmacy PrEP Service to help reduce preventable HIV infection. Respondents agreed that improved accessibility of PrEP from pharmacies is likely to reduce the incidence of new HIV cases, especially as community pharmacies are usually situated within 15–20 min walking distance for most residents in urban areas, have consultation rooms and are familiar with having sensitive conversations such as emergency oral contraception. Providing PrEP in Community Pharmacy was perceived as a way to make preventative HIV treatment more readily accessible compared with current availability.

*“Yes, easily accessible, people need this rather than going to clinic”* (Male Pharmacist p1).

*“There is a big gap in the service. Community pharmacy is confidential and if a client has trust with the Community Pharmacy team, they are more likely to pop in”* (Female Technician p6).

In addition, most respondents considered that despite the current lack of specific training about PrEP, most pharmacists already have the most important competencies required to provide such a service.

*“*…*good consultation skills, empathy”* and *“confidence to speak to clients generally”* (Male Pharmacist p3).

Thus, the majority of interviewees felt that experience gained through other community pharmacy services provide a level of underlying confidence to have a conversation with a patient about sexual health, HIV but this would be enhanced by further topic specific training.

Several respondents also emphasised that pharmacists are knowledgeable about drug interactions and could therefore discuss concurrent medications with clients interested in using PrEP.

*“…knowledge of drug interactions, certain level of education”* (Female Pharmacist p*2*).

One respondent also highlighted that community pharmacies have.

“*information technology (IT) infrastructure and IT competency to use data capture platforms to maintain accurate records”* (Female Pharmacist p2).

### Recommendations

In response to the barriers identified, and building on current strengths, respondents provided several recommendations to support the provision of PrEP in the community pharmacy setting.

*“Important to have rigorous accredited training”* (Female Pharmacist 4).

The availability of training was considered important for staff directly involved in the provision of PrEP, with one participant stating that.

*“accredited training of appropriate staff may include technicians in the process"* (Female Technician p6).

In addition to specific information on PrEP, its dosage and mode of use, respondents suggested that a selection of training should be available to all pharmacy staff interacting with the public, for example.

*“customer service skills training”* for counter staff who are likely to interact first with the patient, because: *“These can be very personal and sensitive issues to discuss, and experiences may put off reattending”* (Female Pharmacist p9).

Along with professional training, respondents noted that public health campaigns for the general public must accompany the provision of such a service.

*“Campaigns to educate on HIV”* and *“raising awareness where they can go for further information, specifically on where they can find it”* (Male Pharmacist p1).

*“Campaigns must be clearly communicated”* (Male Pharmacist p3)*.*

*“Campaigns should explain what HIV is, how it is contracted, prevented and treated and where preventative treatment can be accessed”* (Female Pharmacist p2).

Respondents also highlighted the need for targeted campaigns for disadvantaged and minority groups in places where these groups are likely to socialise or work, with an effort to educate and remove the stigma attached to conversations about HIV infection. These locations may include community centres, religious centres, sports centres, and clubs, using leaflets, posters, and media adverts.

*“*Local advertisements in community magazines, leaflets, posters” (Male Pharmacist p7).

*“Campaign must be clearly communicated, can be general or targeted, for example community centres, religious centres, basically where the clients go”* (Female Pharmacist p2).

These campaigns should also not be limited to English but should be made available in different languages.

*“More information available for everyone and in multiple languages, especially those where English is not the first language. More diversity in literature, if people don't know they don't know"* (Female Pharmacist p9)*.*

Overall sentiment on a Community Pharmacy PrEP supply service:

*“I think Community Pharmacy can play an important role in the introduction of PrEP and increasing awareness of HIV by providing information and consultation to patient”* (Female Technician p10).

## Discussion

### Summary of main findings

The findings from our study provide valuable insights into the perspectives of pharmacy professionals, their attitudes toward PrEP and their recommendations for its successful implementation in community pharmacy settings.

We found that only a minority of community pharmacies offer sexual health services, with varying levels of provision, including chlamydia screening, emergency contraception and HIV testing. Community pharmacies can engage with both NHSE (NHS England) and Local Authority commissioned services or provide elements of sexual health services privately. The majority of survey respondents and interviewees were affiliated to pharmacies in North-West London where they report there is little commissioning of sexual health services. In the UK, women aged over 16 years have been able to purchase progestogen-only emergency hormonal contraception (EHC) from pharmacists without prescription or a dedicated service since 2001. Only a minority of pharmacies stated as already providing some activity related to sexual health services, implying that the sale or supply of EHC may not be perceived by the pharmacy team as part of sexual health services. However, according to both the survey and interviews, and with appropriate training, community pharmacy teams expressed confidence in developing the competencies required to engage in a commissioned Community Pharmacy PrEP service. A new UK NHSE-commissioned advanced Community Pharmacy Contraceptive Service was launched in November 2023, enabling community pharmacists to initiate and maintain supply of oral contraceptive medication. This provides further confirmation that community pharmacists are ready to develop the experience and skills required for new commissioned sexual health services, which could include PrEP in the future.

Further, as of September 2026 newly qualified pharmacists will enter the General Pharmaceutical Council register as independent prescribers and will have the ability to prescribe within their competencies. Although currently Community Pharmacy Independent Prescribers do not have the facility to prescribe within the NHS, the majority of survey respondents who are already or progressing towards becoming an independent prescriber said that they would consider prescribing PrEP in the future.

Without a Community Pharmacy PrEP service, study participants would refer patients requesting PrEP to Sexually Transmitted Infection clinics or suggest online purchase of HIV test kits. While there is moderate knowledge about HIV and PrEP among pharmacists, confidence in providing sexual health services and discussing HIV-related issues varies. The interviewees elaborated that a lack of confidence can be allayed by an accredited training programme to accompany a PrEP service. Although the need for training was considered a barrier to providing a PrEP service, community pharmacists in the UK are familiar with continuing professional development as part of the annual re-registration process, therefore training requirements could be considered a facilitator for providing a PrEP service. Time constraints, and staffing issues, were also raised as barriers, but the study has shown that pharmacists believed these could be overcome by an appropriately funded service. Further, at the time of the survey, only 4.6% of respondents felt uncomfortable discussing sexual health and PrEP and only 1.9% of respondents did not consider offering PrEP as a commissioned service. So, community pharmacy teams’ views and awareness of PrEP should not be perceived as a barrier to providing a Community Pharmacy PrEP service. In fact, within an accredited framework, such a service is likely to be welcomed. Few respondents expressed reluctance to provide a PrEP service on the grounds of stigma, religious beliefs, or fear of infection, and therefore would be unlikely barriers to the provision of a Community Pharmacy PrEP service. The main perceived facilitators were found to be self-expressed competence, confidence, and readiness of community pharmacy teams to engage with a commissioned Community Pharmacy PrEP service.

Many community pharmacies already provide a suite of clinical services, and beyond NWL, this may include a range of sexual health services, building the background experience and skills required for new commissioned activities.

Both survey and interview responses indicated that community pharmacy teams are highly motivated to upscale and engage in clinical services. Addressing perceived barriers such as funding and training would enable pharmacy teams to confidently begin conversations regarding HIV and the availability of community pharmacy provision of PrEP. Just as important is the need for public health awareness campaigns to educate the public about HIV, its prevention and how to access PrEP. Targeted campaigns may help to reduce stigma and cultural sensitivities, encouraging people to come forward for advice. The community pharmacy offers a more accessible, accredited, commissioned PrEP service that would provide improved availability of an important and potentially lifesaving HIV preventative treatment and could help to reduce healthcare inequalities in England.

### Comparison to existing literature

The study's findings align with existing literature on PrEP implementation and HIV prevention. The identified barriers, such as funding and the need for training, are consistent with challenges reported in previous research [25–27]. The knowledge gap among pharmacists regarding PrEP is also in line with concerns raised in the literature about healthcare providers' awareness and knowledge of this preventive measure [25].

A recent scoping review of the Facilitators and Barriers to Community Pharmacy PrEP delivery by Harrison et al. [28] discusses the topic from the perspective of the “capability, opportunity, motivation” model of behaviours (COM-B) and comments that to date no studies have aimed to identify and map the potential barriers and facilitators of PrEP delivery, according to a behavioural theory or model. If the results of this study were to be framed in terms of a COM-B model, the findings would similarly show that pharmacy teams believe that existing knowledge of PrEP (capability) and lack of commissioned service (opportunity) could be considered barriers. However, the expanding number of Community Pharmacy Clinical services provides an example of Community Pharmacy’s readiness and motivation to embrace new challenges. A suitably funded NHS commissioned Community Pharmacy PrEP supply service accompanied by training would improve capability, provide opportunity, and motivate pharmacy teams to engage in this activity.

The emphasis on public awareness campaigns and targeted outreach to disadvantaged and minority populations reflects the broader literature on HIV prevention. Many studies have highlighted the importance of community education and awareness programs to promote HIV prevention methods effectively [25, 29].

Recent endorsements by key stakeholders, as highlighted in a September 2023 article by ‘The Pharmacist’ advocate for the availability of PrEP through community pharmacies, suggesting a unified call for expanded access to this crucial preventive tool [30]. This endorsement by the HIV and AIDS All-Party Parliamentary Group and the Royal Pharmaceutical Society, which emphasises pharmacists' integral role in reducing health inequalities and enhancing patient access to treatment, validates our study's conclusions and recommendations. Our research aligns with the existing body of literature and adds a nuanced understanding of the specific challenges and opportunities within the UK context, particularly in community pharmacy settings. By addressing these identified gaps, our study contributes valuable insights into optimising PrEP provision and HIV prevention strategies.

### Study implications

The implications of this research are significant for both policy and practice. The study highlights the importance of policy initiatives to support the integration of PrEP services in community pharmacy, whereas policymakers should consider funding mechanisms and training programs to overcome identified barriers. Additionally, public health policies should prioritise awareness campaigns, especially in areas where at-risk populations are concentrated. Pharmacy professionals could play a crucial role in HIV prevention, and this research highlights the need for ongoing training and education. Accredited training programs are needed to equip community pharmacists and support staff with the necessary competencies to provide PrEP services. Additionally, community pharmacies should actively engage in public awareness efforts and collaborate with local organisations to reach at-risk populations.

### Study strengths and limitations

To our knowledge, this is the first study that investigated the perspectives of community pharmacy staff regarding the extant barriers and facilitators for the routine provision of HIV PrEP in community pharmacies in the UK, aiming to surface recommendations from community pharmacy staff on how to mitigate these barriers. It was decided that the choice of questions in both the survey and interviews would enable the participants to provide their own views on the barriers and facilitators without seeding suggestions. Therefore, a strength in the study is the views of participants, and particularly those of the interviewees were their own and not suggested. The research was strengthened by the participation of pharmacists and pharmacy technicians, providing a well-rounded perspective on PrEP implementation, whereas the use of semi-structured interviews also allowed for in-depth exploration of participants' attitudes and assumptions, providing rich qualitative data.

The principal limitation in the study was that the majority of participants indicated working in pharmacies in North-Central and North-West London. The results may therefore reflect the specific needs of a multiculturally diverse population, which may differ from other areas in England. Further, 68% of eSurvey respondents were Asian, which likely resulted in their over-representation compared to national data. That said, it has been reported that a large proportion (41% in 2014) of pharmacists registered in Great Britain are from a Black, Asian and minority ethnic BAME background and that BAME pharmacists, particularly those from Asian backgrounds, are more likely to work in the community sector [31]. This observation was supported as recently as March 2022 by data estimating that 43.3% of pharmacists and 19.2% of pharmacy technicians in NHS trusts in England were from a BAME background compared to 18% of the general population of England [32].

The relatively small sample size of our study may also limit the generalisability of the findings, and a larger sample could provide more robust insights. Further, the study relies solely on self-reported data which may be subject to bias, and we acknowledge that participants may provide responses they believe are socially desirable to a topic that may still be associated with some stigma. Finally, the findings may be specific to community pharmacies in the UK and may not be directly applicable to other healthcare settings or countries with different healthcare systems.

## Conclusion

Provision of PrEP from selected community pharmacies across the country could begin to address inequality and inadequacy of PrEP provision and generally improve healthcare for certain underserved communities.

This study has found that community pharmacists and their supporting healthcare teams agree that the supply of PrEP from a community pharmacy could improve access to treatment, address inequalities, help to reduce infection with HIV and generally improve healthcare for certain underserved communities. This supports a report by Public Health England in 2019, The Pharmacy Offer for Sexual Health, Reproductive Health, and HIV—A resource for commissioners and providers, stating “their convenient location and informal environment offer opportunities to improve local access and help reduce health inequalities” [33].

Staff involved with the service should undertake accredited training and adequate funding is needed to ensure an effective service is offered within a specified framework. The fact that, at the very least, most community pharmacies provide contraceptive services, and very often more advanced services such as injections and immunisations, indicates the capability of the profession as a whole and the motivation to “step up services” as needed (for example during the Covid-19 epidemic).

A Community Pharmacy PrEP supply service should be supported by general and targeted sexual health public awareness campaigns with educational content to include disease prevention and how to access PrEP.

## Supplementary Information

Supplementary Material 1.

## Data Availability

The anonymous data that support the findings of this study are available from the corresponding author upon reasonable request. Consent to publish data involving identifying images or other personal or clinical details of participants which could compromise anonymity is not applicable to this manuscript.
