# Supplementary material for: Investigating facilitators and barriers to the routine provision of HIV PrEP in community pharmacies in London
Source: BMC Health Serv Res. 2025 Feb 25;25:312. doi: 10.1186/s12913-025-12336-1 (PMC11863588; doi:10.1186/s12913-025-12336-1)
Supplement: Supplementary file 1 — Supplementary Material 1. [file 12913_2025_12336_MOESM1_ESM.docx]

**Supplementary Files**

**Supplementary File 1: eSurvey export**

Q1
The national rollout of HIV pre-exposure prophylaxis (PrEP) by the NHS is a step in the right direction in aiding the scale-up of PrEP use in the UK, though demand for PrEP remains unmet. Uptake & adherence to PrEP within the community remains largely understudied, and in particular the attitudes & perceptions of pharmacists towards PrEP & its provision in the community pharmacy setting.

Imperial College London Self-Care Academic Unit (SCARU) is conducting a qualitative research study to explore this further. Please take a moment to review the Participant Information Sheet of our study which also has ethical approval in place and consider taking part in this 5-10 min eSurvey. Please feel free to disseminate the study information & link to your personal contacts of pharmacists and health & social care professionals.

Q2 Please confirm that you consent to participate in this survey.

- Yes (1)
- No (2)

Q3 Do you work in a pharmacy?

If you do not work in a pharmacy, please consider taking our short community survey (click here).

- Yes (1)
- No (2)

Q4 What is your role?

- Pharmacist (1)
- Non-pharmacist team member (2)

Q5 Which field(s) do you work in? Please select one or more

1. Community pharmacy (Independent) (1)
2. Community pharmacy (Multiple) (2)
3. Hospital pharmacy (3)
4. GP practice-based pharmacist (4)
5. PCN pharmacist (5)
6. CCG pharmacist (6)
7. Independent prescriber (8)
8. Other (please specify): (7) __________________________________________________


   Q6 Please state the ODS code of the pharmacy you usually work at.

   ________________________________________________________________


   Q7 Are you an independent prescriber?

- Yes (1)
- No (2)
- Working towards IP qualification (3)

Q8 Does your pharmacy provide sexual health services?

- Yes (1)
- No (2)

*Display This Question:*

*If Does your pharmacy provide sexual health services? = Yes*

Q9 Which level of sexual health services do you provide?*

*Click here for a brief description of the services offered at the different levels sexual health services

- Level 1 - STI screening (1)
- Level 2 - testing (2)
- Level 3 - treatment (3)
- Unsure (4)

*Display This Question:*

*If Does your pharmacy provide sexual health services? = Yes*

Q10 What sexual health services do you currently provide? Please select one or more

1. Free condom distribution (1)
2. Emergency hormonal contraception (2)
3. Chlamydia screening/treatment (3)
4. STI testing (4)
5. HIV testing (5)
6. Signposting (8)
7. None of the above (6)
8. Other (please specify): (7) __________________________________________________


   Q11 Which of the following groups are at an elevated risk of HIV?
9. Men who have sex with men (1)
10. ethnic minorities (5)
11. young people (aged 20-24) (6)
12. Sex workers (7)
13. Injecting drug users (4)
14. Other (please specify): (8) __________________________________________________


    Q12 What would you likely do if a patient requested PrEP in your pharmacy?
15. Signpost to an STI clinic (1)
16. suggest they purchase PrEP from safe online websites (4)
17. suggest they proceed with a HIV test (2)


    Q13
    Would you know where to signpost individuals (e.g. to local sexual health clinic/s)?

- Yes (1)
- No (2)

*Display This Question:*

*If Does your pharmacy provide sexual health services? = No*

Q14 If you do not provide any sexual health services, what is the reason?

1. Not needed where I work (1)
2. Need more training (2)
3. Ethical / religious reasons (3)
4. Unsure/ I am not the decision maker (6)
5. Other (please specify): (4) __________________________________________________


   Q15 Have you heard of PrEP (pre-exposure prophylaxis) to prevent HIV?

- Yes (1)
- No (2)

*Display This Question:*

*If Are you an independent prescriber? = Yes*

*Or Are you an independent prescriber? = Working towards IP qualification*

Q16 If you are an independent prescriber, or are working towards an IP qualification, would you consider prescribing PrEP?

- Yes (1)
- No (2)

Q17 To what extent do you agree with the following statements?

|  | 1. Strongly disagree (1) | 1. Disagree (2) | 1. Neither agree nor disagree (3) | 1. Agree (4) | 1. Strongly agree (5) |
| --- | --- | --- | --- | --- | --- |
| 1. I feel comfortable signposting patients to HIV & sexual health services (9) |  |  |  |  |  |
| 1. I feel confident to provide private/commissioned sexual health services in my pharmacy (10) |  |  |  |  |  |
| 1. I feel confident to discuss issues regarding patients' sexual health (11) |  |  |  |  |  |
| 1. I feel confident to discuss HIV health issues/services with a patient (12) |  |  |  |  |  |
| 1. I am not/would not be worried about the HIV status of patients using pharmacy HIV services (13) |  |  |  |  |  |
| 1. I would feel more comfortable/confident providing services if I had more training or support (14) |  |  |  |  |  |
| 1. It would be appropriate if individuals can access PrEP from the community pharmacy setting (1) |  |  |  |  |  |
| 1. Individuals will be comfortable to receive health information from their local community pharmacy regarding PrEP (2) |  |  |  |  |  |
| 1. Making PrEP available in the community pharmacy setting will raise awareness & demand towards PrEP in the community (4) |  |  |  |  |  |
| 1. I routinely signpost patients to STI clinics (7) |  |  |  |  |  |

Q18 Would you consider offering a commissioned service to supply PrEP if full training were provided?

- Yes (1)
- Unsure/ I am not the decision maker (3)
- No (please explain why): (2) __________________________________________________

Q19 What would prevent you from offering PrEP in pharmacy? Please select one or more

1. Time needed to counsel individuals (4)
2. Require more training (5)
3. Insufficient staffing levels (9)
4. Uncomfortable discussing sexual matters (6)
5. Other (Please specify) (8) __________________________________________________


   Q20 What is your gender?

- Male (1)
- Female (2)
- Other (please specify) (3) __________________________________________________
- Prefer not to say (4)

Q21 How old are you? (In years)

________________________________________________________________

Q22 Which borough do you work in?

________________________________________________________________

Q23 Choose an option which best describes your ethnic background:

- English/Welsh/Scottish/Northern Irish/British (1)
- Irish (2)
- Gypsy or Irish Traveller (3)
- Any other White background, please describe (4) __________________________________________________
- White and Black Caribbean (5)
- White and Black African (6)
- White and Asian (7)
- Any other Mixed/Multiple ethnic background, please describe (8) __________________________________________________
- Indian (9)
- Pakistani (10)
- Bangladeshi (11)
- Chinese (12)
- Any other Asian background, please describe (13) __________________________________________________
- African (14)
- Caribbean (15)
- Any other Black/African/Caribbean background, please describe (16) __________________________________________________
- Arab (17)
- Any other ethnic group, please describe (18) __________________________________________________

Q24 As a note of gratitude, we are rewarding all recipients a £15 Amazon voucher following completion of the survey. if you would like to receive this voucher, please enter your email below.

________________________________________________________________

Q25 Thank you for taking the time to complete our survey.

Researchers from Imperial College London are looking to interview up to 30 participants (via telephone, Skype or Microsoft Teams) to learn more about your experiences involving PrEP. Interviews will last 25-35 minutes (and your time will be compensated). Please provide your name and contact details below if this interests you. We are happy to answer any questions about the study & can fix a suitable time and date for an interview.

- Name (1) __________________________________________________
- Email (2) __________________________________________________
- Phone number (3) __________________________________________________

**End of Block: Default Question Block**

**Supplementary File 2: Interview Topic Guide**

| **#** | **Question** |
| --- | --- |
| 1 | Do you think the introduction of PrEP in Community Pharmacy will aid in reducing HIV incidence? |
| 2 | What do you think can be done to meet a high demand for PrEP? |
| 3 | What competencies do you feel are needed by pharmacy staff to streamline the routine provision of PrEP? |
| 4 | What do you think can be done to raise awareness about PrEP within the community? How about for BAME groups or those who are at high risk of contracting HIV but are less likely to take PrEP? |
| 5 | What can be done to reach out to groups who are at high risk of HIV and are unaware or unable to access PrEP? |
| 6 | What can be done to raise awareness of PrEP with an emphasis towards minority groups? |

**Supplementary File 3: Interview Table of Themes**

Code: Male-M ; Female-F ; Pharmacist-P ; Technician-T ; participant number- p = (1-10)

| **Driver / Barrier** | **Sub Theme** | **Specific Points** | **Comments** |
| --- | --- | --- | --- |
| **Drivers** | Enthusiasm Of Community Pharmacy Team | All interviewees were positive about community Pharmacy provision of PrEP | ***FPp2*** *"Yes, if it were readily available and advertised as commissioned service”,*  ***MPp2*** *"Yes, easily accessible, people need this rather than going to clinic"* |
|  | Accessibility of Community Pharmacies | Most people in urban areas are within a 20-minute walk from a community pharmacy | ***MPp3*** *"Yes, improved access from community pharmacy will help"…"* |
|  | Competencies of Staff | Many Community Pharmacies are already providing a range of services and staff are required to be trained to high standard. Accredited training for a PrEP service will be required but is seen as a Driver not a barrier. To provide certain services, Community Pharmacist is required to have completed relevant consultation skills training and good communication skills. | ***FPp2*** *"Consultation skills, empathy, knowledge of drug interactions, certain level of education, IT competency to use data capture platforms, also accredited training"*  ***FTp6*** *"accredited training of appropriate staff ie may include technicians in the process"*  ***FPp4*** *“good communication skill, accredited training”*  ***MPp5*** *“consultation skills, lot of accredited training”*  ***FTp10*** *“ability to professionally approach patient”* |
|  | Confidentiality | Most Pharmacies have a consultation room | ***FTp6*** *“advertising campaigns, need to remove stigma and make people feel more comfortable about coming forward. More likely to come in (to pharmacy) if run a campaign including (that pharmacy has) confidential consultation rooms* etc |
| **Barriers** | Lack of public Awareness of HIV and PrEP | Public awareness campaigns, both national and targeted towards different communities, are needed to raise awareness of the availability of PrEP | ***FPp9*** *"More information in multiple languages, especially those where English is not the first language. More diversity in literature, if people don't know they don't know****”***  ***MPp3 “****Publicise where client can get it, Community Pharmacy ?"* |
|  | Lack of education | Lack of general education around sexual health and protection from disease. | ***MPp1*** *“ are they aware”…”it’s a big taboo around what it is and the risks”*  ***MPp3*** *“education on safe sex, raise the profile of the condition so more people are aware”* |
|  | Stigma around HIV & sex | PrEP users may be sensitive about using the service due to shyness, stigma, religious beliefs | ***FPp9*** *“People in different communities may have issues sharing experiences and not wanting people to know”…”More privacy needed for one-on-one sessions at the Pharmacy”*    *Different cultures have different needs, we need to make people feel safe and able to talk." .... "Taboo subject for some cultures and families"*  ***FTp6*** "*need to remove stigma"* *and make people feel more comfortable about coming forward."* |
|  | Education & awareness | Not all healthcare staff aware of the availability of PrEP referenced in survey | ***MPp5*** *"Need to provide education about HIV, how it can be caught and how to prevent and treat. Sexual health promotion”*  ***FPp2*** *“media, promotional, educational campaigns where these minorities frequent…to come to community pharmacy if treatment becomes available”* |
|  | Funding | Community Pharmacy will need a fully funded NHS commissioned PrEP supply service | ***MPp1****"* *a barrier is* *funding, in light of recent contraception service this is important."*  *FPp8 “the biggest issue is money to facilitate a service”* |
|  | Lack of confidence & discomfort with the topic | Some interviewees were concerned about the lack of confidence around "having the conversation" but this would be addressed with training | ***FPp4*** "*Embarrassment, staff and client"*  ***MPp5*** *"concern about (PrEP Service) potentially may increase promiscuity”*  ***FPp9*** “*Education Training required for staff, these can be very personal and sensitive issues to discuss”* |
|  | Lack of Time | the issue of making time during busy workflow was mentioned as is often the case with something new, but this issue has not deterred many other service implementations. | ***FTp10*** *“not enough time to counsel patient as may be needed”*  ***FTp6*** " *(a barrier is) pharmacist time but could help if service extended to capable technicians."* |
| **Recommendations** | Community Pharmacists and technicians are overwhelmingly in favour of providing PrEP but within an accredited framework which will include appropriate training to support the service and enhance the confidence of the pharmacy staff involved at any given stage of the service process. | A community Pharmacy PrEP provision service needs to be properly funded via NHS commissioning. This will address inequalities regarding access to PrEP | A national Public Health campaign alongside targeted campaigns is needed to improve sexual health knowledge generally as well as HIV specifically, how the infection is transmitted, prevented, treated and where to access services. Important to help reduce stigma and sensitivities surrounding discussions about HIV. |
